# Fetal Eye Movements on Magnetic Resonance Imaging

**DOI:** 10.1371/journal.pone.0077439

**Published:** 2013-10-23

**Authors:** Ramona Woitek, Gregor Kasprian, Christian Lindner, Fritz Stuhr, Michael Weber, Veronika Schöpf, Peter C. Brugger, Ulrika Asenbaum, Julia Furtner, Dieter Bettelheim, Rainer Seidl, Daniela Prayer

**Affiliations:** 1 Department of Biomedical Imaging and Image-guided Therapy, Medical University of Vienna, Vienna, Austria; 2 Center for Anatomy and Cell Biology, Medical University of Vienna, Vienna, Austria; 3 Department of Obstetrics and Gynecology, Medical University of Vienna, Vienna, Austria; 4 Department of Paediatrics and Adolescent Medicine, Medical University of Vienna, Vienna, Austria; University of Pécs Medical School, Hungary

## Abstract

**Objectives:**

Eye movements are the physical expression of upper fetal brainstem function. Our aim was to identify and differentiate specific types of fetal eye movement patterns using dynamic MRI sequences. Their occurrence as well as the presence of conjugated eyeball motion and consistently parallel eyeball position was systematically analyzed.

**Methods:**

Dynamic SSFP sequences were acquired in 72 singleton fetuses (17–40 GW, three age groups [17–23 GW, 24–32 GW, 33–40 GW]). Fetal eye movements were evaluated according to a modified classification originally published by Birnholz (1981): Type 0: no eye movements; Type I: single transient deviations; Type Ia: fast deviation, slower reposition; Type Ib: fast deviation, fast reposition; Type II: single prolonged eye movements; Type III: complex sequences; and Type IV: nystagmoid.

**Results:**

In 95.8% of fetuses, the evaluation of eye movements was possible using MRI, with a mean acquisition time of 70 seconds. Due to head motion, 4.2% of the fetuses and 20.1% of all dynamic SSFP sequences were excluded.

Eye movements were observed in 45 fetuses (65.2%). Significant differences between the age groups were found for Type I (p = 0.03), Type Ia (p = 0.031), and Type IV eye movements (p = 0.033). Consistently parallel bulbs were found in 27.3–45%.

**Conclusions:**

In human fetuses, different eye movement patterns can be identified and described by MRI in utero. In addition to the originally classified eye movement patterns, a novel subtype has been observed, which apparently characterizes an important step in fetal brainstem development. We evaluated, for the first time, eyeball position in fetuses. Ultimately, the assessment of fetal eye movements by MRI yields the potential to identify early signs of brainstem dysfunction, as encountered in brain malformations such as Chiari II or molar tooth malformations.

## Introduction

During prenatal human brain development, the brainstem reaches a relatively high grade of maturity [1], [2]. Due to the small dimensions of the fetal midbrain, prenatal morphologic evaluation is still difficult. Since the complex neuronal network that governs human eye movements is mainly located in the midbrain, the assessment of fetal eye motion may open a diagnostic window to the detection of abnormal midbrain maturation and function. Currently, our knowledge about the patterns and characteristics of human fetal eye movements is based on ultrasound studies only [3], [4], and limited mainly by the orbital bones and the fetal position in utero.

Recently, it has been shown that fetal magnetic resonance imaging (MRI) might feasibly provide time-resolved dynamic imaging data about the intrinsic and gross fetal movements in real-time from 18 gestational weeks (GW) onward [5]. MRI provides standardized dynamic images of the fetal ocular bulbs in different planes, presumably allowing a more objective evaluation and classification of fetal eye movements.

The aim of this fetal MRI study was, first, to assess whether fetal eye movements can be reliably demonstrated using dynamic MR sequences, and second, to investigate whether different types of eye movements can be distinguished non-invasively in order to acquire more detailed knowledge about the functional maturation of the neuronal networks involved in oculomotor control. The third aim was to study the time of onset of different eye movement patterns, and fourth, to examine the position of the two fetal ocular bulbs relative to each other. More detailed knowledge about eye movements in normally developing fetuses may be a starting point for the evaluation of eye movements in fetuses suffering from brain malformations that primarily involve or affect the midbrain, such as molar tooth malformations, Chiari II malformations, or hydrocephalus.

## Patients and Methods

### Ethics statement and consent

The study was approved by the Ethics Committee of the Medical University of Vienna and conducted according to the Declaration of Helsinki. Written, informed consent to obtain additional dynamic sequences of the eyeballs was obtained in advance.

### Patients

All 72 patients were referred to our department for fetal MRI between 2008 and 2010 to rule out or to confirm suspicious findings on fetal ultrasound.

The established gestational age was based on ultrasound examinations during the first trimester. Birnholz divided the fetuses examined in his ultrasound study into three age groups: 17–23 GW, 24–32 GW, and 33–40 GW, but did not further explain his specific motivations for that. Our classification of different types of eye movements is mainly based on Birnholz's study. We wanted our data to be comparable to the results of Birnholz and other authors [3], [4], [6] and therefore, we decided to divide fetuses into the same age groups as previously described.

MRI revealed no pathology (n = 28) or isolated congenital pathologies that did not affect the CNS (n = 44). The most common pathologies were congenital diaphragmatic hernia in five fetuses, gastroschisis in four fetuses, cleft lip and palate in three fetuses, and esophageal atresia in three fetuses.

### MRI

MRI examinations were performed on a 1.5 Tesla system (Philips Medical Systems, Best, The Netherlands) with a five-element, phased-array cardiac coil. No contrast agents or sedation were used.

The MRI protocol included dynamic steady-state free precession (SSFP) sequences in the coronal, axial, or sagittal orientation (FOV: 300 mm, matrix: 256/256, slice thickness: 12–30 mm, TR: 3096–3252 ms, TE: 1564–1624 ms, flip angle 90°, duration: 35 s, 210 slices, 6 slices per second), static axial and coronal T2-weighted ultrafast spin-echo sequences, and axial and coronal SSFP sequences, acquired as described in a previous publication [7], and adjusted according to the changing structural composition of the fetal brain during gestation.

### Image evaluation

**Figure 1:**
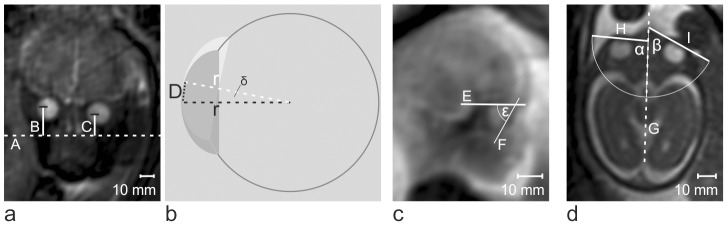
Examples of coronal, *a*, sagittal, *c*, and axial, *d*, images at 34+4GW, 21+1GW, and 26 GW respectively and the lines drawn to measure bulb positions and to calculate bulb deviations. **a** Coronal frame of a dynamic MR sequence. A horizontal line at the level of the most caudal part of the hyperintense nasal choanae is drawn, *A*. Vertical line at the vertical axis of both lenses are drawn separately, *B* and *C*, craniocaudally and perpendicularly to the horizontal line A. **b** Schematic drawing of the eyeball on two sequential frames with the bulb radius on these frames forming an isoscles triangle and allowing the calculation of the angle δ of bulb deviation. (Details concerning the calculation of the angles of bulb deviations between sequential coronal frames can be found in the Methods section.) **c** *S*agittal frame of a dynamic MR sequence. Two lines were marked as references to indicate the hypointense roof of the orbit, *E*, and the longest axis of the sagittally depicted lens, *F*. The angle ε enclosed by these two lines was measured and differences between the angle ε on sequential frames were calculated. *d* shows an axial frame of a dynamic MR sequence. Three lines were marked as references to indicate the intracranial midline, *G*, and the longest axis of the axially depicted lens in each eye, *H* and *I*. The angle enclosed by the line through the longest axis of the lens and the intracranial midline was measured on each side, *α* and *β*.

Measurements were performed on coronal, sagittal, and axial images. In the following paragraph, the evaluation processes for all image orientations are described in detail. A visual description can be found in Figure 1.

On each **coronal image**, three different lines were added to indicate reference lines: A horizontal line at the level of the most caudal part of the hyperintense nasal choanae (Figure 1a, dotted line A), and at the vertical axes of both lenses separately at their widest parts, craniocaudally and perpendicularly to the horizontal line A (Figure 1a, line B and C). For each side, the distance between the center of the lens and the horizontal line A was subsequently measured along the craniocaudal lines B and C. The distances by which the center of the lens had moved on consecutive frames were calculated.

The angle δ by which a bulb had moved between two frames was then calculated using an isosceles triangle, as shown in Figure 1b. In this triangle, the radius (Figure 1b, black r) of the eyeball in one frame (perpendicular to the long axis of the lens) constitutes one of the two equal sides enclosing the angle δ. The radius of the eyeball on the next frame (Figure 1b, white r) constitutes the second side of the triangle. The base of the triangle is the distance (Figure 1b, D) between the position of the center of the lens in the first frame and in the next frame. The radius (Figure 1b, r) of the bulb was measured on axial images of each fetus. As the radius, r, and the distance, D, between the center points of the lens in one frame and in the subsequent frame are known, the angle δ of the isosceles triangle can be calculated as follows:


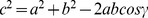
 or 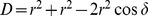, with 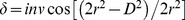.

On **sagittal images**, two lines were marked as references to indicate the hypointense roof of the orbit (Figure 1c, line E), and the longest axis of the sagittally depicted lens (Figure 1c, line F). The angle ε enclosed by these two lines, E and F, was measured and differences between the angle ε on sequential frames were calculated.

On **axial sequences**, three lines were marked as references to indicate the intracranial midline (Figure 1d, line G), and the longest axis of the axially depicted lens in each eye (Figure 1d, H and I). The angle enclosed by the line through the longest axis of the lens and the intracranial midline was measured on each side (Figure 1d, α and β) on subsequent frames.

Differences between the angles α on sequential frames, as well as between the angle β on sequential frames, were calculated. The difference between an angle on two subsequent frames (Δ°) was then divided by the time interval between two image frames (0.167 seconds). The result of this division was defined as the speed of eye movement in degrees per second (°/s).

A radiologist (R.W.) assessed fetal eye movements using a modified classification originally devised by Birnholz (1981) for ultrasound examinations, and described in more detail by Bridgeman (1983) (Table 1) [3], [6]. Birnholz defined Type I eye movements as single, transient deviations consisting of a bulb deviation and a slower return back to the resting position[3]. Later, Bridgeman explained Type I movements as active bulb deviations, with an inability to hold the bulb in an eccentric position so that the bulb drifts back to the resting position passively[6]. Based on our observations of two different types of bulb deviations and repositions, we split up Type I eye movements into Type Ia (fast deviation, slower reposition) and Type Ib (fast deviation, equally fast reposition). Birnholz further classified single but prolonged eye movements as Type II, complex sequences of eye movements to different directions without periodicity as Type III, and repetitive nystagmoid eye movements as Type IV. We further added the criteria of at least three multidirectional deviations to the definition of Type III and of at least three unidirectional eye movements to Type IV. Three or more repetitive unidirectional eye movements on one dynamic sequence (frequency ≥0.43 Hz) were classified as Type IV, independent of their velocity, as they represent nystagmoid eye movements rather than a real nystagmus according to Birnholz's own study [3]. No eye movements were classified as Type 0 in this study.

**Table 1:** Modified classification of fetal eye movements based on a classification made by Birnholz [3] using ultrasound findings.

				all fetuses (%)		17–23 GW (%)		24–32 GW (%)		33–40 GW (%)	
**Type 0**		no eye movements		24	(34.8)	7	(43.8)	12	(36.4)	5	(25)
**Type I**		single transient deviation		14	(20.3)	6	(37.5)	6	(18.2)	1	(5)
	**Type Ia**		fast deviation, slower reposition	7	(10.1)	4	(25.0)	3	(9.1)	0	(0)
	**Type Ib**		fast deviation, equally fast reposition	7	(10.1)	2	(12.5)	4	(12.1)	1	(5)
**Type II**		single persistent deviation		21	(30.4)	2	(12.5)	10	(30.3)	9	(45)
**Type III**		complex sequence of deviations, non-periodic		15	(21.7)	1	(6.3)	9	(27.3)	5	(25)
**Type IV**		repetitive or nystagmoid deviations		7	(10.1)	0	(0)	2	(6.1)	5	(25)

In 11 fetuses, two different types of eye movement were observed.

Bulb positions were evaluated on static, axial T2-weighted sequences, and on static and dynamic axial SSFP sequences. On axial images, the longest axes of the two lenses were evaluated (lines H and I on Figure 1d), and were considered parallel when they enclosed an angle ≤10°. On coronal images they were considered parallel when the distances between the center of one lens and the superior/medial/lateral orbital wall differed ≤1 mm between the left and right eye. Bulb positions were classified as consistently parallel when bulbs remained in a parallel position during an entire dynamic axial or coronal sequence (35 seconds) or on two consecutive axial or coronal static sequences.

### Exclusion criteria

Sequences with severe fetal head movements were excluded from evaluation. If the lens could not be delineated well enough to determine its center on two or more sequential coronal frames, or to determine its longest axis on axial and sagittal frames, the entire sequence was excluded from analysis. If the lens could not be delineated well enough in only one frame, the single frame was excluded and the time interval from the previous slide until the successive image was calculated.

### Statistical evaluation

Differences between the fetal age groups for different types of eye movements or bulb positions were calculated using a two-tailed Fisher's exact test. P-values at or below 0.05 were considered statistically significant. Statistical analyses were performed using the Statistical Package for the Social Sciences, Version 17 (SPSS, Chicago, Illinois).

## Results

Dynamic SSFP sequences were acquired in 72 fetuses (17–40 GW; mean 29±5.6 GW). Seventeen of the examined fetuses were in the youngest age group (17–23 GW; mean 21.7±1.5 GW), 35 fetuses were in the middle age group (23–32 GW; mean 28.4±2.4 GW), and 20 fetuses were in the oldest age group (33–40 GW; mean, 36.1±1.8 GW) (Table 2).

**Table 2:** Descriptive statistics of examined fetuses.

age group (GW)	included fetuses	mean gestational age (GW)
17–23	16	21.7±1.5
24–32	33	28.4±2.4
33–40	20	36.1±1.8
**Total**	**69 (95.8%)**	

In 51 of 72 fetuses (72.2%), dynamic SSFP sequences in the axial (88 sequences), coronal (51 sequences), or sagittal orientation (47 sequences) were acquired intentionally to depict eye movements. In 21 of the 72 fetuses (27.8%), sagittal dynamic SSFP sequences were acquired to depict movements other than that of the eyes, such as swallowing and cardiac or extremity movements. In these 30 sagittal sequences, the eyes were depicted with sufficiently high quality to evaluate eye movements; therefore, these 21 fetuses were included in our study (Table 3).

**Table 3:** Descriptive statistics of dynamic SSFP MRI sequences.

		number		Included (%)	Excluded (%)	eye movements observed (%)
***fetuses***			*72*	*69 (95.8)*	*3 (4.2)*	*45 (65.2)*
**axial dynamic SSFP sequences**			88	68 (77.3)	20 (23.5)	17 (19.3)
**coronal dynamic SSFP sequences**			51	39 (76.5)	12 (23.5)	17(33.3)
**sagittal dynamic SSFP sequences**	acquired to depict eye movements	47	77	43 (91.5)	4 (13.3)	28 (36.4)
	retrospectively included	30				

Of the 88 axial sequences, 20 (23.5%) were excluded due to insufficient image quality (see Exclusion criteria), as well as 12 of the 51 coronal sequences (23.5%), and four of the 30 sagittal sequences (13.3%) that were intentionally acquired for the assessment of eye movements. Of the 72 fetuses, three (4.3%) had to be excluded entirely from the study, as none of the sequences were evaluable due to severe head motion (21+3 GW, 28 GW, 31+1 GW); thus, datasets of 69 fetuses (95.7%) were included in the study (29±5.6 GW).

In two of the included axial sequences, and in seven of the included coronal sequences, only one eye was depicted; in all the other included axial and coronal sequences, both eyes were depicted.

In 17 of the 88 axial sequences (19.3%), eye movements were observed, as well as in 17 of the 51 coronal sequences (33.3%) and in 28 of the 77 sagittal sequences (36.4%). In one of the axial sequences, only one of the two depicted eyes showed movements, and in all the other included axial and coronal sequences, the observed eye movements were conjugated, including both eyes.

Eye movements were found in 45 fetuses (65.2%) during a mean observational period of 98 seconds per fetus (35 seconds per dynamic SSFP sequence, mean: 2.8, range: 1–8 dynamic SSFP sequences per fetus).

Coronal, axial, and sagittal sequences were analyzed quantitatively to evaluate the temporal and spatial resolution of dynamic SSFP sequences with regard to their ability to differentiate between slow and fast eye movements.

**Figure 2:**
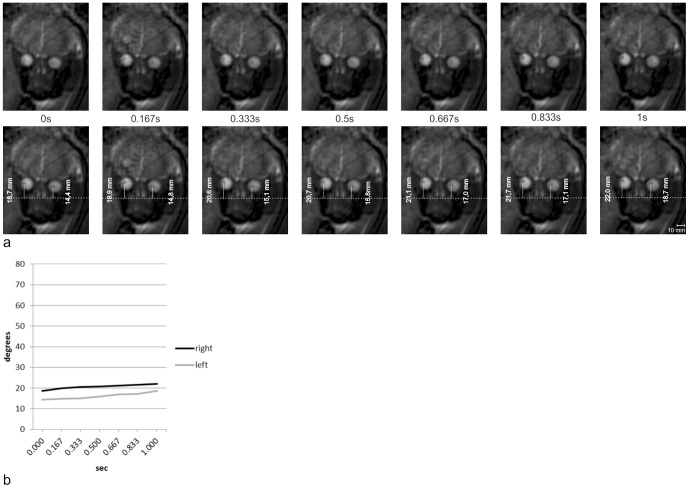
Measurements of bulb positions on sequential coronal images. **a** Seven sequential frames of a coronal dynamic SSFP sequence in a fetus at 34+4 GW (upper row) and the same frames with measurements of bulb positions (lower row). **b** Based on the measurements of bulb positions shown in *Figure 2a*, bulb deviations were calculated and plotted against time. Slow binocular deviations of 3.3° of the right and 4.3° of the left eyeball on seven sequential frames at an average speed of 3.3°/s (right) and 4.3°/s (left). See *Table S1* for complete data on measurements and angular velocities.

**Figure 3:**
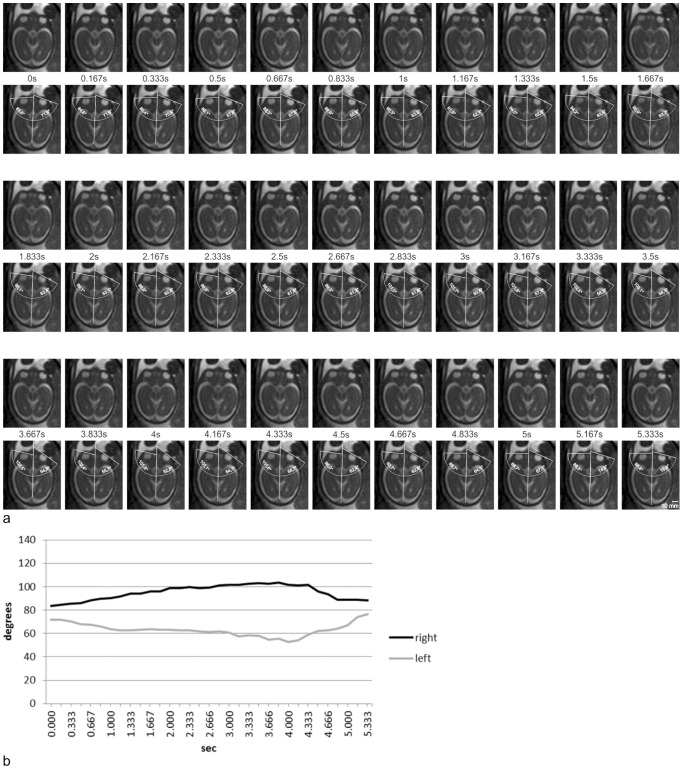
Measurements of bulb positions on sequential axial images. **a** Thirty-three sequential frames of an axial dynamic SSFP sequence in a fetus at 26 GW (first, third, and fifth rows) and the same frames with measurements of bulb positions (second, fourth, and sixth rows). **b** Bulb deviations measured on the 33 sequential axial frames shown in *Figure 3a* were calculated and plotted against time. Initial slow binocular deviation with lateral movement of one eyeball and medial movement of the other eyeball, with amplitudes of 19.7° and 13.6°, respectively, at average velocities of 5.1°/s and 4.1°/s (0.00–4.00 s). Subsequently, the eyeballs were repositioned more rapidly at average velocities of 9.9°/s and 9.1°/s (4.00–5.33 s). See *Table S2* for complete data on measurements and angular velocities.

**Figure 4:**
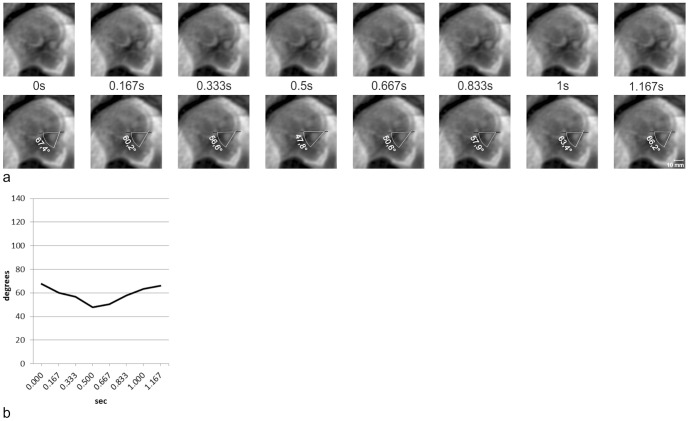
Measurements of bulb positions on sequential sagittal images. **a** Eight sequential frames of a sagittal dynamic SSFP sequence in a fetus at 21+1 GW (upper row) and the same frames with measurements of bulb positions (lower row). **b** Bulb deviations on eight sequential sagittal frames shown in *Figure 4a* were calculated and plotted against time. Craniocaudal deviation of one sagittally depicted eyeball with an amplitude of 19.6° at an average velocity of 39°/s, and the subsequent reposition at an average velocity of 37°/s. See *Table S3* for complete data on measurements and angular velocities.

**Figure 5:**
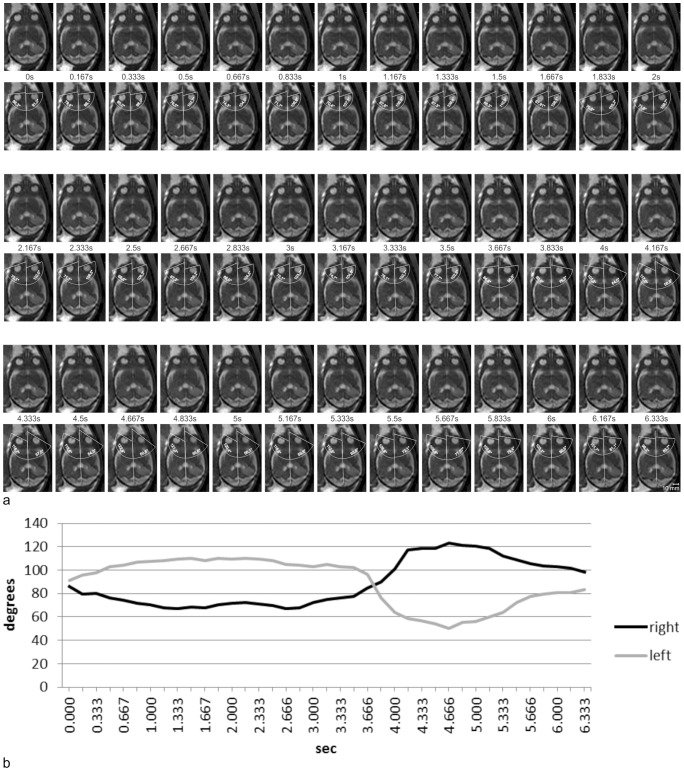
Measurements of bulb positions on sequential axial images. **a** Thirty-nine sequential frames of an axial dynamic SSFP sequence in a fetus at 27+1 GW (first, third, and fifth rows) and the same frames with measurements of bulb positions (second, fourth, and sixth rows). **b** Bulb deviations on the 39 sequential axial frames shown in *Figure 5a* were calculated and plotted against time. Initially, this fetus showed a slow binocular version (0–2.17 s) with amplitudes of 14.2° and 19.1° of the right and left eye, respectively, at velocities of 6.6°/s and 8.8°/s. Subsequently, the eyeballs were repositioned (2.17–3.67 s) at average velocities of 8.3°/s (right) and 9.0°/s (left). A second version follows (3.67–4.67 s) with a fast initial component at average velocities of 65°/s and 75.8°/s for the right and the left eyeballs respectively. The second part of this deviation decreased in average to 11.8°/s and 16.2°/s respectively. The entire amplitudes of these biphasic deviations were 38.4° and 46.0°, with average velocities of 38.4°/s and 46°/s for the right and the left eyeball, respectively. The eye movements in this sequence are among the fastest observed in this study. The right bulb shows a deviation of 15.7° between 4.00 s and 4.17 s (94°/s), and the left bulb shows a deviation of 20.2° between 3.83 s and 4.00 s (121.0°/s). From 4.67–6.33 s, a slow reposition of both eyeballs at velocities of 14.8°/s (right) and 20.0°/s (left) follows. See *Table S4* for complete data on measurements and angular velocities.

Quantitative analysis of exemplary dynamic axial, coronal, and sagittal SSFP sequences revealed that eye movements of different velocities and directions could be distinguished. Slow eye movements, at velocities between 3.3°/s and 19.6°/s (Figure 2 and 3), and fast eye movements peaking at a velocity of 121°/s, have been observed (Figures 4 and 5). Sample eye movements have been analyzed quantitatively and are shown in Figures 2–5. Complete quantitative data on angles of deviation and velocities are shown in supplementary Tables S1–S4.

Among all fetuses, Type 0 eye movements (no eye movements) were observed in 24 fetuses (34.8%). Type I eye movements were observed in 14 fetuses (20.3%). Type I eye movements are defined as a bulb deviation and subsequent reposition [3] (Table 1). Because the reposition is passive, it is slower than the preceding bulb deviation [6]. We observed bulb repositions slower than the preceding deviation (Figure 5, 3.67–6.33 s), as well as repositions as fast as or faster than the deviation (Figure 3); therefore, we further distinguished between Type Ia (deviation, slower reposition) and Type Ib eye movements (deviation, reposition of at least equal or higher velocity).

**Figure 6:**
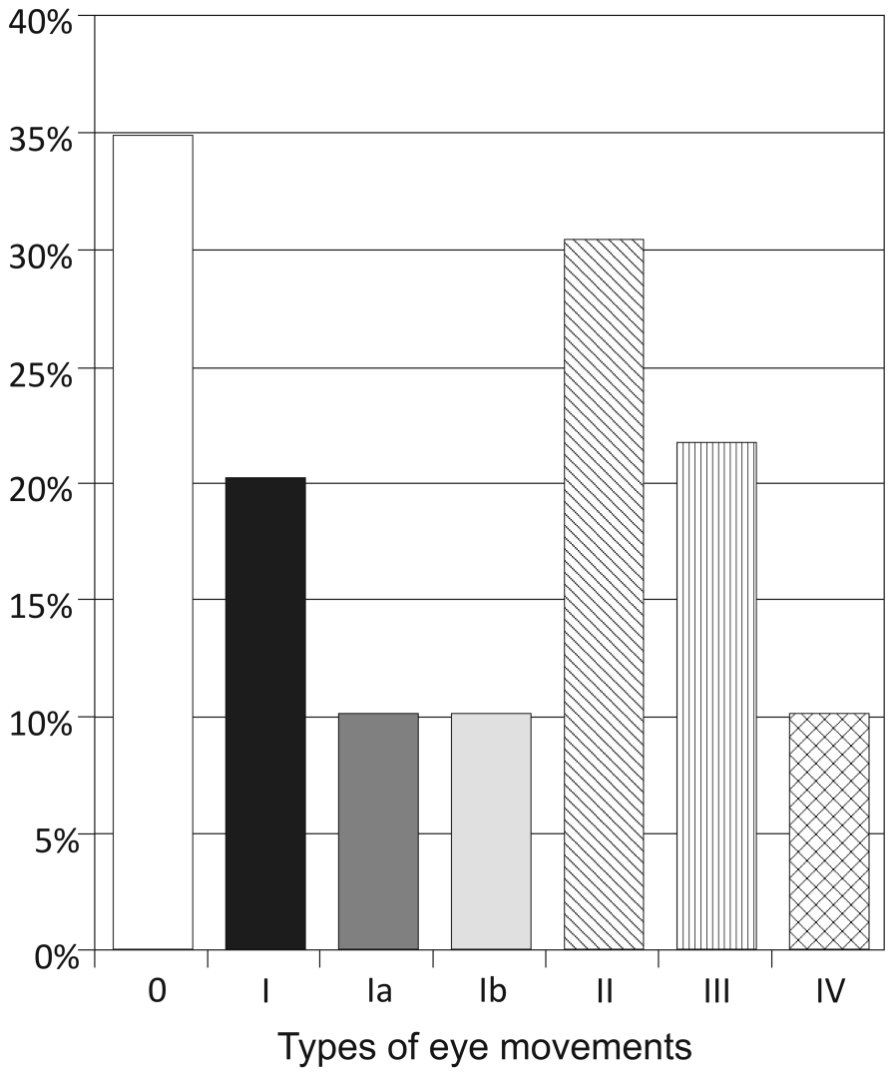
Prevalence of different types of eye movements among all fetuses. In 11 fetuses (15.9%), two different types of eye movements were observed.

Type Ia movements were observed in seven fetuses (10.1%) and Type Ib movements in seven fetuses (10.1%). Type II movements were observed in 21 fetuses (30.4%), Type III in 15 fetuses (21.7%), and Type IV in seven fetuses (10.1%) (Figure 6).

In 11 fetuses (15.9%), two different types of eye movements were observed. One fetus showed Types Ib and II (1.4%), one fetus showed Types Ia and III (1.4%), six fetuses showed Types II and III (8.7%), and three fetuses showed Types II and IV eye movements (4.3%).

Type Ia eye movements were found in the youngest fetuses from 17–23GW (4/16 fetuses, 25%), and in fetuses from 24–32 GW (3/33 fetuses, 9.1%), whereas Type Ib movements were found up until 34 GW, and thus, were included in the age group from 33–40GW (1/20 fetuses 5%).

**Figure 7:**
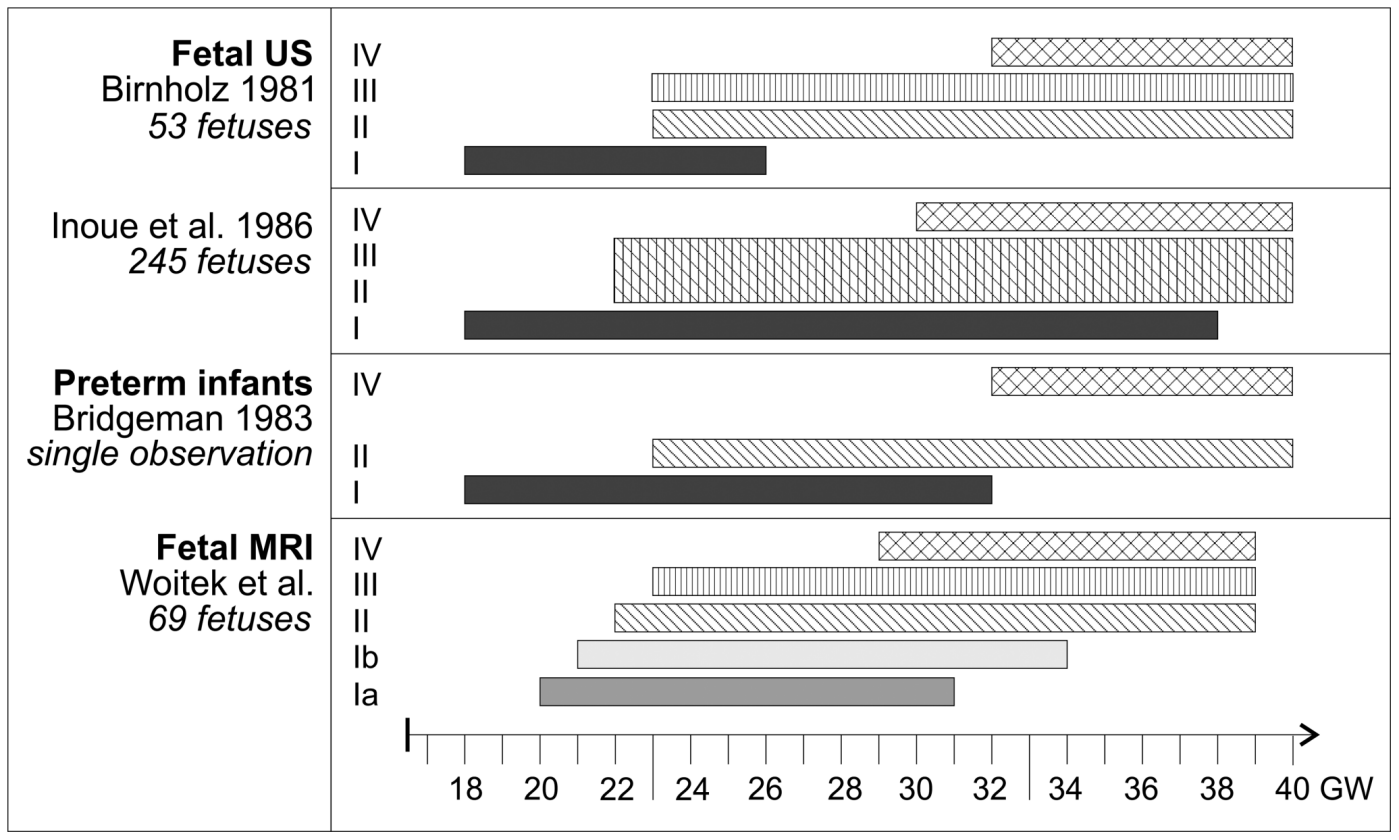
Intervals during which different types of eye movements could be observed in this study and in previous reports on prenatal ultrasound examinations [3], [4] and in premature newborns [6].

Type II and III eye movements started to occur at similar time-points, with Type II at 23 GW and Type III at 22 GW, and both continued until 40 GW. Type IV occurred at later gestational ages, beginning at 29 GW (Figure 7).

**Figure 8:**
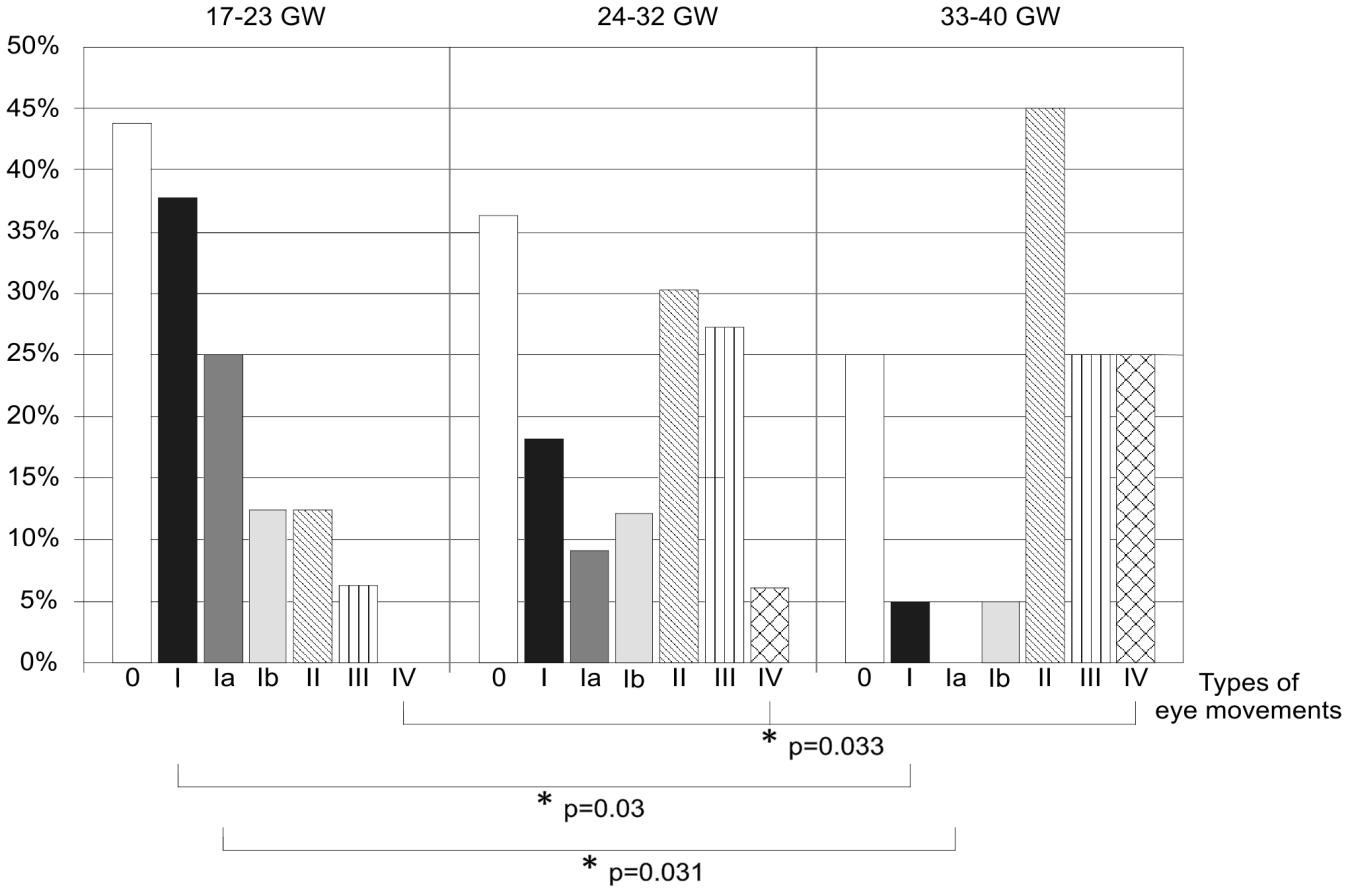
Prevalence of different types of eye movements in three fetal age groups. Asterisks and brackets indicate those types of eye movements in which significant differences were found.

Significant differences concerning Type I eye movements (p = 0.03) and Type Ia movements (p = 0.031) were found between the youngest and the oldest age groups. For Type IV eye movements, a significant difference was found when comparing all three age groups (p = 0.033) and when comparing the youngest and oldest age groups (p = 0.053, slightly above the chosen level of significance) (Figure 8).

Bulb position could be evaluated in 39 of the 69 included fetuses (56.5%). In the remaining 30 fetuses (43.5%), bulb position could not be evaluated sufficiently because both lenses were not shown equally in one axial or coronal slice.

Of 69 fetuses, 24 showed consistently parallel bulbs (34.8%), and 15 of 69 did not show parallel bulb positions on the acquired slices (21.7%).

We found consistently parallel bulbs in fetuses of all age groups and found this feature earliest in fetuses as young as 18 GW and 20+5 GW.

**Figure 9:**
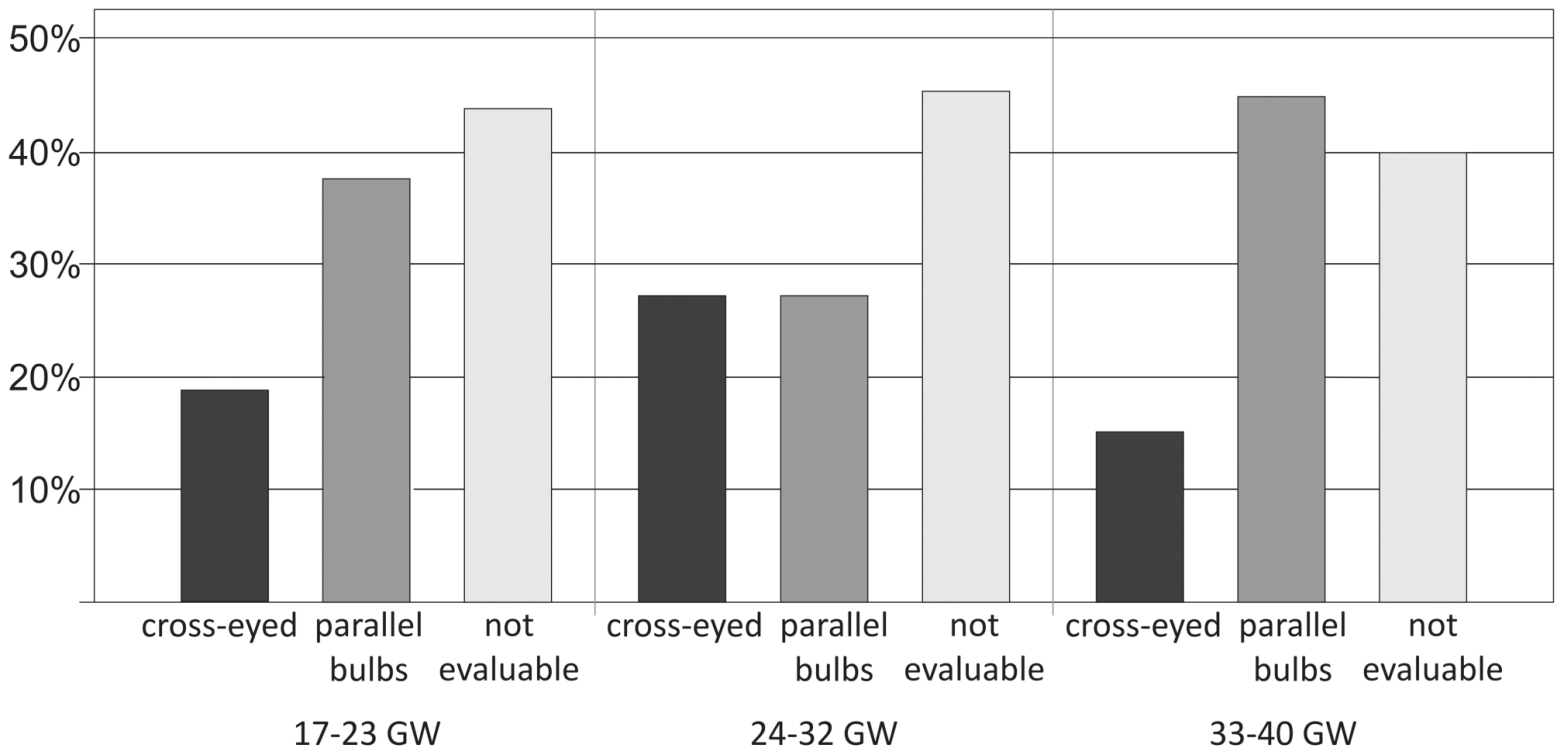
Prevalence of parallel bulb position and cross-eyed condition, as well as lack of images depicting both lenses to evaluate bulb position.

Consistently parallel bulbs were found in nine of 20 fetuses from 33–40 GW (45%), in nine of 33 fetuses from 24–32GW (27.3), and in six of 16 fetuses from 17–23GW (37.5%) (Figure 9). Differences between the fetal age groups were not significant (p = 0.717).

## Discussion

In our study to evaluate fetal eye movements in a population of 72 fetuses, the evaluation of eye movements was possible using dynamic SSFP MR sequences in 95.8% of all the examined fetuses. Severe head motion or inadequate depiction of the lenses caused the exclusion of 4.2% of fetuses. These difficulties could be overcome in the majority of cases with repetitions of dynamic SSFP sequences (up to eight per fetus). We showed that dynamic MR imaging of the fetal eyes is technically feasible. Additional dynamic sequences only slightly prolong the MR examination, so that the overall examination time of 45 minutes was well tolerated [8].

Observational periods of 30 seconds [3] -110 minutes [4] have been reported in ultrasound studies, with eye movements observed in 62%–100%. The observational period of 98 seconds and the prevalence of eye movements of 65.2% in our MR study are comparable to these values.

We were able to show, for the first time, that dynamic MR sequences are a valuable tool to examine, demonstrate, and quantitatively evaluate fetal eye movements. Furthermore, the temporal and spatial resolution of MRI enable the differentiation between fast and slow eye movements, as demonstrated in Figures 2–5, showing velocities of bulb deviations ranging from 3.3°/s to 75.8°/s, maintained over several frames and peaking as high as 121.0°/s between two single frames. Amplitudes measured on two sequential frames ranged from 0.1° to 20.2°. Due to the spatial resolution of dynamic MRI sequences angular readings between 0.1°–0.5° cannot be sufficiently differentiated from noise, and therefore, are not considered as movement due to measurement uncertainty.

In a study of healthy infants, the mean ***smooth pursuit*** gain (eye velocity divided by the stimulus velocity used) between 1–3 months of age was below 0.5 at stimulus velocities of 7.5°–30°/s. The mean velocity of smooth pursuit was therefore 3.5°–15°/s, although maxima of smooth pursuit gains of 1–1.2 were observed (7.5–45°/s) [9]. In a study of smooth pursuit on healthy full-term newborns, eye movement velocities were found below 15°/s, and, most often, even below 10°/s [10]. In the presented study we showed that slow eye movements at velocities similar to those of newborns or young infants can be observed in fetuses using dynamic MRI sequences. Furthermore, we show that dynamic MRI sequences can even be used to differentiate between slow eye movements of different velocities.

***Fast eye movements*** were observed at velocities between 300°/s and 600°/s, with amplitudes between 4° and 30° in a study of infants 2 to 18 months of age [11]. In another study of infants 14–151 days of age, saccadic velocity ranged from 130°/s to 300°/s for an amplitude of 10° [12]. The highest velocity observed in our study was 121.0°/s and was cearly lower than the velocities reported in infant saccades. The reasons for this discrepancy might be the ongoing maturation of the oculomotor system until birth and thereafter. Clearly, different settings might also contribute, as, in our study, exclusively spontaneous eye movements are examined, whereas, in the above-mentioned studies saccades or nystagmus evoked by visual stimuli were analyzed, which might have caused higher velocities.

The temporal course of different types of eye movements in our study is compared to previously published works on fetal ultrasound and premature infants in Figure 7. Inoue et al. classified frequencies of fetal eye movements as ‘low’, ‘moderate’, and ‘high’ [4]. Low frequency corresponded to Birnholz's Type I, moderate frequency to Types II and III, and high frequency to Type IV. Due to the limited observation time, the assessment of frequencies of eye movements was beyond the scope of this study. The time-points of onset and disappearance of types of eye movements in our study are similar to those in previously published data [3], [4], [6].

Eleven fetuses showed two different types of eye movements. A statistical analysis concerning the prevalence of several different types of eye movements exhibited by single fetuses was not performed, as the number of those fetuses was too small. The finding indicates that eye movements in one fetus are not limited to one single type but can include sequential movements that fulfill the criteria for several types of eye movements, one type at a time.

The advantages of dynamic MRI of the fetal eyeballs over ultrasound are less restriction by the orbital bones, and by the position of the fetal head, and higher soft tissue contrast of the bulbar structures.

Type I eye movements were previously defined as a bulb deviation and a slower reposition [3], [6]. We observed eye movements of precisely this pattern, but also observed eye movement patterns consisting of a deviation and an equally fast reposition. As the reposition was not slower than the deviation, it was not necessarily consistent with Bridgeman's description of a passively driven reposition [6], but could have represented active movement. Therefore, we separated the two movement patterns into Type Ia (fast deviation, slower reposition) and Type Ib (fast deviation, equally fast reposition). The prevalence of Type I and Type Ia movements was significantly higher in the youngest group than in the oldest age group (p = 0.03 and 0.031). At the same time, there was an increase in the prevalence of Type II movements from the youngest to the oldest fetal age group, although it did not reach significance (p = 0.114).

It is known that the saccadic system (paramedian pontine reticular formation, rostral interstitial medial longitudinal fascicle) generates bulb deviations, whereas the neuronal integrator of the oculomotor system (nucleus prepositus hypoglossi, medial vestibular nucleus, interstitial nucleus of Cajal) generates the active maintenance of an eccentric bulb position [13]. As a result of the ability to keep the eye in an eccentric position, the prevalence of Type Ia eye movements decreases over time. Separating single bulb deviations according to the speed of reposition, and thus, supposedly passive or active repositioning, enables the observer to distinguish between important developmental steps in the brainstem.

We observed a tendency of Type II eye movements to increase linearly with gestational age (Figure 8), although the differences between the three age groups do not reach the level of significance. Nevertheless, we consider this tendency an important finding as it might be an expression of the increasing ability to maintain eccentric bulb positions and maturation of the neuronal integrator of the oculomotor system. With examinations of even larger cohorts, the increasing prevalence of Type II eye movements with gestational age might reach the level of significance and might, therefore, prove this conclusion more reliably.

Confirming the results of previous studies, we found Type III eye movements at earlier gestational ages than Type IV [3], [4], [6]. It has been shown that fetal behavioral states develop from the second trimester of pregnancy, but only become distinguishable from 36 GW onward, based on fetal heart rates, as well as body and eye movements [14], [15]. The behavioral states, 1F and 2F, have been equated functionally to fetal non-REM sleep and REM-sleep, respectively [14], [16], and both Type III and IV eye movements have also been reported to be associated with REM sleep [3], [6]. Although occurring as early as at 22 and 29 GW, Type III and IV eye movements may represent maturation of the complex neuronal interactions within the pontine reticular formation in which REM-sleep is generated by that gestational age [17]. Our results are also consistent with findings in premature infants showing that REM and non-REM sleep can be distinguished by at least 10 GW [18].

Diurnal rhythms of human fetal activity states have not been evaluated in detail. In a study of baboon fetuses at 80–90% of term age, 28.5% of the time was spent in an identifiable behavioral state, with 29.1% of this time spent in state 1F [19]. With lights turned on during the day, the prevalence of the 1F state reached 12.7%. Because in state 1F no continuous eye movements are usually observed (contrary to other behavioral states), we expected that, for at least 12.7% of the entire observational time, we would not be able to observe any continuous eye movements. In a study by Pillai and James (1990) of human fetuses between 36 and 42 GW, 30.2% of the observational time was identified as state 1F, regardless of diurnal rhythm [20].

We were not able to detect eye movements in 34.8% of our fetuses. This result might partly be attributable to fetal behavioral state 1F, but state 1F might only account for the inability to observe movements up to 30.2% of the time, as described above. Furthermore, many of the fetuses in our study were at younger gestational ages than those in the above-mentioned studies, possibly accounting for the lower prevalence of eye movements. In addition, with longer observational periods, we might have detected eye movements in more fetuses. To evaluate whether the time-point of examination reveals differences in fetal eye movements, and to evaluate the behavioral state of the fetuses, were beyond the scope of this study, but should be part of future investigations.

This study is the first to investigate the parallel position of the eyeballs prenatally. The highest prevalence of consistently parallel bulbs was found in the oldest age group, which might be indicative of maturational processes of the oculomotor system. However, the differences between the three age groups were not significant, nor could any tendency be observed toward a linear increase in the prevalence of parallel bulbs with age. In addition, the fact that bulb positions could not be evaluated in 43.5% of fetuses because both lenses were not shown exactly in one axial or coronal slice emphasizes the limitations of this study. Because of the small size of fetal eyeballs [9.97 mm at 18 GW [21]] and lenses, the evaluation of their positions relative to each other is particularly challenging. If one of the two lenses is cut centrally by the imaging plane, and the other lens is cut peripherally, the position may be inaccurately perceived as a cross-eyed position. However, the small-sized bulbs and lenses might make convergent or divergent bulbs less apparent.

**Figure 10:**
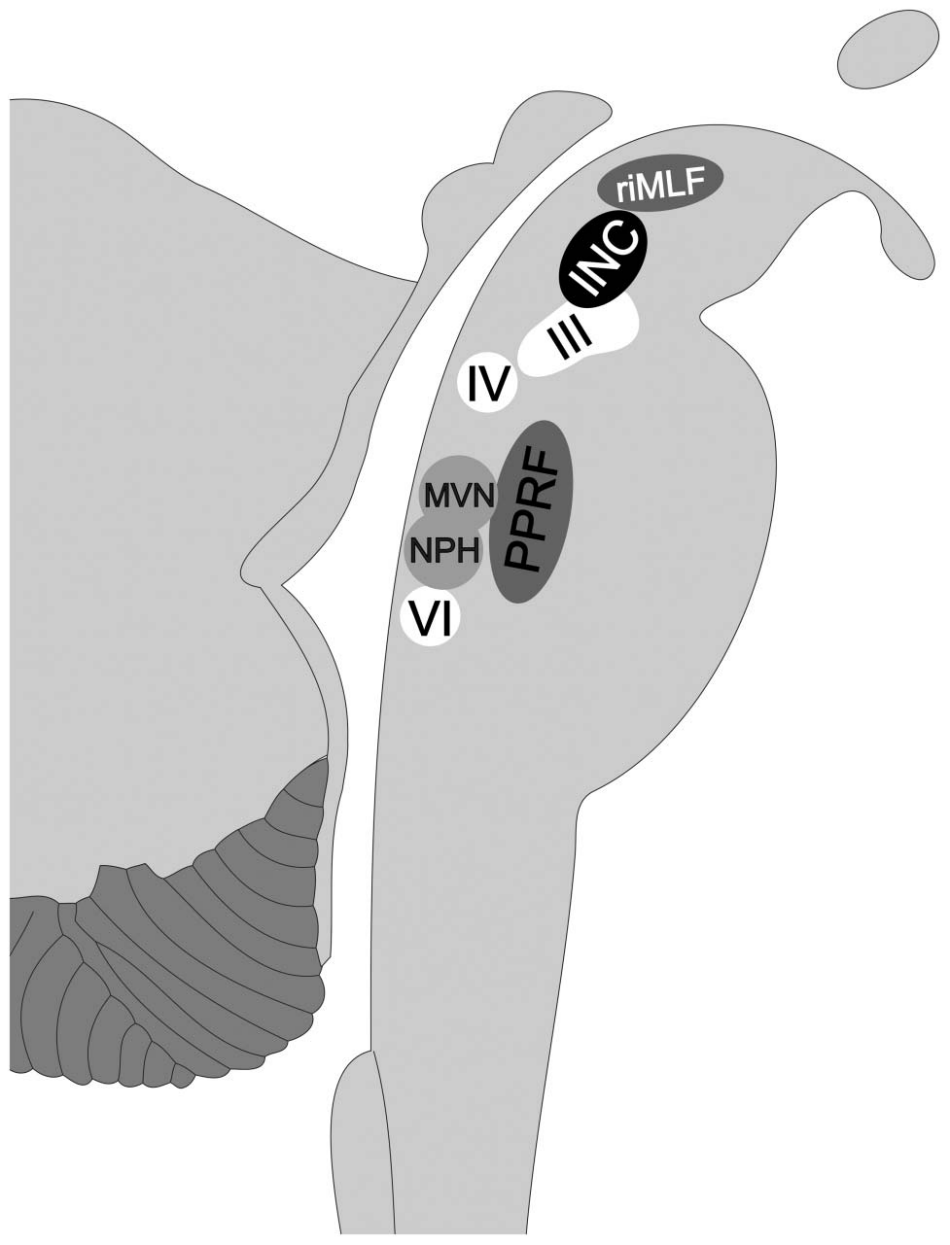
Structures of the brainstem responsible for the generation of eye movements in fetuses. riMLF =  rostral interstitial nucleus of the medial longitudinal fasciculus; INC =  interstitial nucleus of Cajal; III =  nucleus of the third cranial nerve; (oculomotor nerve); IV =  nucleus of the fourth cranial nerve (trochlear nerve); VI =  nucleus of the sixth cranial nerve (abducent nerve); PPRF =  paramedian pontine reticular formation; MVN =  medial vestibular nucleus; NPH =  nucleus prepositus hypoglossi.

Our study proves, for the first time, that MRI is a valuable tool for the visualization of fetal eye movements. Our findings confirm previously published results about different types of eye movements occurring at different gestational ages, and, for the first time, distinguish between passive and active repositioning of the eyeballs from an eccentric position, shedding light on an important step of brainstem maturation. For the clinician, our findings might serve as an additional imaging biomarker, especially in fetuses with malformations that affect the brainstem. For example, in fetuses with molar tooth malformations, hydrocephalus, or Chiari malformations, the presence of the described eye movements might indicate integrity of structures such as the oculomotor, trochlear, the abducent nucleus, the neuronal integrator, and the saccadic system (Figure 10).

## Supporting Information

Table S1 Quantitative data on right and left eyeball position in a 34+4 GW old fetus, measured on sequential frames of the coronal dynamic SSFP sequence shown in *Figure 2a*.
(DOCX)

Table S2 Quantitative data on right and left eyeball position in a 26 GW old fetus, measured on sequential frames of the axial dynamic SSFP sequence shown in *Figure 3a*.
(DOCX)

Table S3 Quantitative data on eyeball position in a 21+1 GW old fetus, measured on sequential frames of the coronal dynamic SSFP sequence shown in *Figure 4a*.
(DOCX)

Table S4 Quantitative data on right and left eyeball position in a 27+1 GW old fetus, measured on sequential frames of the axial dynamic SSFP sequence shown in *Figure 5a*.
(DOCX)
