# Supplementary material for: Fetal Eye Movements on Magnetic Resonance Imaging
Source: PLoS One. 2013 Oct 23;8(10):e77439. doi: 10.1371/journal.pone.0077439 (PMC3806733; doi:10.1371/journal.pone.0077439)
Supplement: Table S1 — Quantitative data on right and left eyeball position in a 34+4 GW old fetus, measured on sequential frames of the coronal dynamic SSFP sequence shown in Figure 2a . (DOCX) [file pone.0077439.s001.docx]

| **Right eyeball** | | | | **Left eyeball** | | | |
| --- | --- | --- | --- | --- | --- | --- | --- |
| sec | position (°) | Δ° | °/s | sec | position (°) | Δ° | °/s |
| 0.000 | 18.7 |  |  | 0.000 | 14.4 |  |  |
| 0.167 | 19.9 | 1.2 | 8.6 | 0.167 | 14.8 | 0.4 | 2.9 |
| 0.333 | 20.6 | 0.7 | 5 | 0.333 | 15.1 | 0.3 | 2.1 |
| 0.500 | 20.7 | 0.1 | 0.7 | 0.500 | 15.8 | 0.7 | 5.0 |
| 0.667 | 21.1 | 0.4 | 2.9 | 0.667 | 17.0 | 1.2 | 8.6 |
| 0.833 | 21.7 | 0.6 | 4.3 | 0.833 | 17.1 | 0.1 | 0.7 |
| 1.000 | 22.0 | 0.3 | 2.1 | 1.000 | 18.7 | 1.6 | 1.0 |
