# Supplementary material for: Fetal Eye Movements on Magnetic Resonance Imaging
Source: PLoS One. 2013 Oct 23;8(10):e77439. doi: 10.1371/journal.pone.0077439 (PMC3806733; doi:10.1371/journal.pone.0077439)
Supplement: Table S2 — Quantitative data on right and left eyeball position in a 26 GW old fetus, measured on sequential frames of the axial dynamic SSFP sequence shown in Figure 3a . (DOCX) [file pone.0077439.s002.docx]

| **Right eyeball** | | | | **Left eyeball** | | | |
| --- | --- | --- | --- | --- | --- | --- | --- |
| sec | position  (°) | Δ° | °/s | sec | position (°) | Δ° | °/s |
| 0.000 | 83.8 |  |  | 0.000 | 71.9 |  |  |
| 0.167 | 84.6 | 0.8 | 4.9 | 0.167 | 71.6 | -0.3 | -1.8 |
| 0.333 | 85.4 | 0.8 | 5.0 | 0.333 | 70.4 | -1.2 | -7.2 |
| 0.500 | 86.1 | 0.7 | 4.1 | 0.500 | 67.8 | -2.6 | -15.6 |
| 0.667 | 88.3 | 2.2 | 13.2 | 0.667 | 67.5 | -0.3 | -1.8 |
| 0.833 | 89.9 | 1.6 | 9.5 | 0.833 | 65.9 | -1.6 | -9.6 |
| 1.000 | 90.3 | 0.5 | 2.7 | 1.000 | 63.5 | -2.4 | -14.4 |
| 1.167 | 91.9 | 1.5 | 4.0 | 1.167 | 62.7 | -0.8 | -4.8 |
| 1.333 | 94.0 | 2.1 | 12.7 | 1.333 | 62.6 | -0.1 | -0.6 |
| 1.500 | 94.2 | 0.2 | 1.2 | 1.500 | 63.4 | 0.8 | 4.8 |
| 1.667 | 95.9 | 1.7 | 9.9 | 1.667 | 63.7 | 0.3 | 1.8 |
| 1.833 | 96.1 | 0.3 | 1.5 | 1.833 | 63.4 | -0.3 | -1.8 |
| 2.000 | 99.0 | 2.9 | 17.2 | 2.000 | 63.1 | -0.3 | -1.8 |
| 2.167 | 98.8 | -0.2 | 1.2 | 2.167 | 62.8 | -0.3 | -1.8 |
| 2.333 | 99.6 | 0.8 | 4.7 | 2.333 | 62.6 | -0.2 | -1.2 |
| 2.500 | 98.9 | -0.7 | 4.1 | 2.500 | 61.6 | -1.0 | -6.0 |
| 2.667 | 99.3 | 0.4 | 2.5 | 2.667 | 61.3 | -0.3 | -1.8 |
| 2.833 | 101.0 | 1.7 | 10.2 | 2.833 | 61.6 | 0.3 | 1.8 |
| 3.000 | 101.7 | 0.7 | 4.2 | 3.000 | 60.6 | -1.0 | -6.0 |
| 3.167 | 101.8 | 0.1 | 0.5 | 3.167 | 57.3 | -3.3 | -19.8 |
| 3.333 | 102.5 | 0.7 | 4.3 | 3.333 | 58.3 | 1.0 | 6.0 |
| 3.500 | 103.2 | 0.7 | 3.9 | 3.500 | 58.1 | -0.2 | -1.2 |
| 3.667 | 102.5 | -0.7 | 4.1 | 3.667 | 54.8 | -3.3 | -19.8 |
| 3.833 | 103.4 | 1.0 | 5.8 | 3.833 | 55.7 | 0.9 | 5.4 |
| 4.000 | 101.8 | -1.6 | -9.7 | 4.000 | 52.8 | -2.9 | -17.4 |
| 4.166 | 101.1 | -0.8 | -4.5 | 4.166 | 54.1 | 1.3 | 7.8 |
| 4.333 | 101.7 | 0.6 | 3.5 | 4.333 | 58.7 | 4.6 | 27.5 |
| 4.500 | 96.0 | -5.7 | -33.9 | 4.500 | 62.4 | 3.7 | 22.2 |
| 4.667 | 93.8 | -2.2 | -13.4 | 4.667 | 62.6 | 0.2 | 1.2 |
| 4.833 | 88.7 | -5.1 | -30.5 | 4.833 | 64.1 | 1.5 | 9.0 |
| 5.000 | 88.7 | 0.0 | 0.2 | 5.000 | 67.1 | 3.0 | 18.0 |
| 5.167 | 88.7 | 0.0 | 0.1 | 5.167 | 74.0 | 6.9 | 41.3 |
| 5.333 | 88.6 | -0.1 | -0.8 | 5.333 | 76.4 | 2.4 | 14.4 |
