# Supplementary material for: Fetal Eye Movements on Magnetic Resonance Imaging
Source: PLoS One. 2013 Oct 23;8(10):e77439. doi: 10.1371/journal.pone.0077439 (PMC3806733; doi:10.1371/journal.pone.0077439)
Supplement: Table S3 — Quantitative data on eyeball position in a 21+1 GW old fetus, measured on sequential frames of the coronal dynamic SSFP sequence shown in Figure 4a . (DOCX) [file pone.0077439.s003.docx]

| sec | position (°) | Δ° | °/s |
| --- | --- | --- | --- |
| 0.00 | 67.4 |  |  |
| 0.167 | 60.2 | 7.2 | 43.1 |
| 0.333 | 56.6 | 3.6 | 21.6 |
| 0.5 | 47.8 | 8.8 | 52.7 |
| 0.667 | 50.6 | -2.8 | -16.8 |
| 0.833 | 57.9 | -7.3 | -43.7 |
| 1.00 | 63.4 | -5.5 | -32.9 |
| 1.167 | 66.2 | -2.8 | -16.8 |
