# Supplementary material for: Fetal Eye Movements on Magnetic Resonance Imaging
Source: PLoS One. 2013 Oct 23;8(10):e77439. doi: 10.1371/journal.pone.0077439 (PMC3806733; doi:10.1371/journal.pone.0077439)
Supplement: Table S4 — Quantitative data on right and left eyeball position in a 27+1 GW old fetus, measured on sequential frames of the axial dynamic SSFP sequence shown in Figure 5a . (DOCX) [file pone.0077439.s004.docx]

| **Right eyeball** | | | | **Left eyeball** | | | |
| --- | --- | --- | --- | --- | --- | --- | --- |
| sec | position  (°) | Δ° | °/s | sec | position (°) | Δ° | °/s |
| 0.000 | 86.3 |  |  | 0.000 | 91.0 |  |  |
| 0.167 | 79.4 | -6.9 | -41.3 | 0.167 | 96.1 | 5.1 | 30.5 |
| 0.333 | 80.0 | 0.6 | 3.6 | 0.333 | 98.0 | 1.9 | 11.4 |
| 0.500 | 76.5 | -3.5 | -21.0 | 0.500 | 102.7 | 4.7 | 28.1 |
| 0.667 | 74.3 | -2.2 | -13.4 | 0.667 | 104.0 | 1.3 | 7.8 |
| 0.833 | 71.7 | -2.6 | -15.3 | 0.833 | 106.6 | 2.6 | 15.6 |
| 1.000 | 70.4 | -1.3 | -7.8 | 1.000 | 107.7 | 1.1 | 6.6 |
| 1.167 | 67.9 | -2.5 | -14.9 | 1.167 | 108.5 | 0.8 | 4.8 |
| 1.333 | 67.3 | -0.6 | -3.5 | 1.333 | 109.4 | 0.9 | 5.4 |
| 1.500 | 68.3 | 1.0 | 5.7 | 1.500 | 110.0 | 0.6 | 3.6 |
| 1.667 | 67.9 | -0.4 | -2.3 | 1.667 | 108.1 | -1.9 | -11.4 |
| 1.833 | 70.6 | 2.7 | 16.0 | 1.833 | 110.3 | 2.2 | 13.1 |
| 2.000 | 71.9 | 1.3 | 7.9 | 2.000 | 109.7 | -0.6 | -3.5 |
| 2.167 | 72.1 | 0.2 | 1.2 | 2.167 | 110.1 | 0.4 | 2.2 |
| 2.333 | 71.1 | 1.4 | 8.6 | 2.333 | 109.5 | -0.6 | -3.4 |
| 2.500 | 69.6 | -3.9 | -23.5 | 2.500 | 108.3 | -1.2 | -7.2 |
| 2.667 | 67.3 | -2.3 | -13.7 | 2.667 | 105.2 | -3.1 | -18.6 |
| 2.833 | 68.0 | 0.7 | 4.1 | 2.833 | 104.5 | -0.7 | -4.2 |
| 3.000 | 72.1 | 4.1 | 24.6 | 3.000 | 103.0 | -1.5 | -9.0 |
| 3.167 | 74.7 | 2.6 | 15.6 | 3.167 | 104.6 | 1.6 | 9.6 |
| 3.333 | 76.1 | 1.4 | 8.4 | 3.333 | 102.7 | -1.9 | -11.4 |
| 3.500 | 77.7 | 1.6 | 9.6 | 3.500 | 102.5 | -0.2 | -1.2 |
| 3.667 | 84.5 | 6.8 | 40.7 | 3.667 | 96.5 | -6.0 | -35.9 |
| 3.833 | 90.0 | 5.5 | 32.9 | 3.833 | 76.3 | -20.2 | -121.0 |
| 4.000 | 101.3 | 11.3 | 67.7 | 4.000 | 64.0 | -12.3 | -73.7 |
| 4.166 | 117.0 | 15.7 | 94.0 | 4.166 | 58.6 | -5.4 | -32.3 |
| 4.333 | 118.7 | 1.7 | 10.2 | 4.333 | 57.0 | -1.6 | -9.6 |
| 4.500 | 118.7 | 0.0 | 0.0 | 4.500 | 54.3 | -2.7 | -16.2 |
| 4.667 | 122.9 | 4.2 | 25.1 | 4.667 | 50.5 | -3.8 | -22.8 |
| 4.833 | 121.3 | -1.6 | -9.6 | 4.833 | 55.5 | 5.0 | 29.9 |
| 5.000 | 120.5 | -0.8 | -4.8 | 5.000 | 56.1 | 0.6 | 3.6 |
| 5.167 | 118.6 | -1.9 | -11.4 | 5.167 | 59.7 | 3.6 | 21.6 |
| 5.333 | 112.3 | -6.3 | -37.7 | 5.333 | 63.7 | 4.0 | 24.0 |
| 5.500 | 108.9 | -3.4 | -20.4 | 5.500 | 72.1 | 12.7 | 76.0 |
| 5.667 | 105.8 | -3.1 | -18.6 | 5.667 | 77.5 | 8.7 | 52.1 |
| 5.833 | 103.7 | -2.1 | -12.6 | 5.833 | 79.4 | -3.1 | -18.6 |
| 6.000 | 103.2 | -0.5 | -3.0 | 6.000 | 80.5 | -1.5 | -9.0 |
| 6.167 | 101.7 | -1.5 | -9.0 | 6.167 | 81.1 | 0.6 | 3.6 |
| 6.333 | 98.2 | -3.5 | -21.0 | 6.333 | 83.7 | 2.6 | 15.6 |
